# A Prospective Study of Villous Capillary Lesions in Complicated Pregnancies

**DOI:** 10.1155/2014/193925

**Published:** 2014-11-24

**Authors:** Anu Priyadharshini Srinivasan, B. O. Parijatham Omprakash, Kandhimalla Lavanya, Priyadharshini Subbulakshmi Murugesan, Saraswathi Kandaswamy

**Affiliations:** ^1^Department of Pathology, Sree Balaji Medical College and Hospital, Bharath University, Chennai, Tamil Nadu 600 044, India; ^2^Department of Obstetrics and Gynaecology, Sree Balaji Medical College and Hospital, Bharath University, Chennai, Tamil Nadu 600 044, India

## Abstract

The vascularity of placental tissue is dependent on various factors of which fetomaternal hypoxia plays a major role. Hypoxia can be of different types and each type influences the vascularity of the villi, especially terminal villi, in its own way. In this study, we attempted to identify villous vascular changes in a group of term placentae from mothers with diseases complicating pregnancy. Chorangiosis was the most frequently identified lesion while chorangioma was found in only 2 cases. There were no cases of chorangiomatosis. A few cases had normal villous vasculature. Maternal diseases have a major role in disrupting the placental vasculogenesis and angiogenesis by creating a hypoxic environment that may affect the fetus adversely. Hence, such conditions need to be identified early in pregnancy and managed appropriately as it is possible to maintain a normal vasculature and prevent neonatal mortality and morbidity if prompt intervention is done.

## 1. Introduction

Placenta is a unique organ derived from both the mother and the fetus and is of prime importance throughout the period of gestation. It is a highly vascular organ whose vasculature is an intricately and tightly regulated complex of vessels from both the mother and the developing fetus. Vasculogenesis and angiogenesis are triggered by numerous growth factors in the molecular microenvironment. Any change that affects the hemodynamics of the maternal blood flow can seriously affect the growth of the placenta and indirectly that of the fetus. Similarly, any change in the hemodynamics of fetal blood flow can also affect the placental growth. Thus, placenta plays a very important role in maintaining homeostasis in both lives during pregnancy.

A good antenatal period with adequate nutrition and absence of systemic diseases is essential to achieve this goal. In few unfortunate patients, maternal diseases may adversely affect the growth of the placenta and in turn that of the offspring. Many studies have been done worldwide to unravel the mysteries of the placenta and to add evidence to the existing clinicopathological correlation studies. In our study, we attempt to find one such evidence at the microscopic level relating to villous vasculature and to classify the lesions based on past literature.

## 2. Materials and Methods

A total of 62 placentae were taken for this prospective study. The study group comprised 31 placentae from complicated pregnancies and control group included 31 placentae from normal pregnancies. The control group consisted of antenatal women with adequate hemoglobin values, blood glucose, and blood pressure within normal limits and with no other symptoms characteristic of disease pathology. The study group included placentae from 10 antenatal women with anaemia, 10 with preeclampsia, 6 with gestational diabetes, and 5 with hypothyroidism. The gestational age of the mothers and the birth weights of their newborn infants are listed in Tables 1 and 2. All women were delivered at term either by normal vaginal delivery or by caesarean section at the Department of Obstetrics and Gynaecology. The delivered placentae were immediately placed in adequate volume of 10% neutral buffered formalin and brought to the Department of Pathology for grossing. After examining the placentae in the fresh state for morphometry and gross abnormalities, the placentae were allowed to fix adequately before sectioning. Representative sections were taken after thorough examination of the specimen for further processing and subjected to hematoxylin and eosin staining. The microscopic features were analyzed and results tabulated.

Grossly, no changes were identified macroscopically and focal changes were clear only in histopathological sections microscopically. Chorangiosis was diagnosed based on the criteria laid down by Altshuler [1]. A few cases showed increased vascularity of the terminal villi but did not satisfy Altshuler's criteria and hence could not be classified as chorangiosis. In case of chorangiomas, gross identification was possible subchorionically in both cases and both showed a reddish brown tinge measuring about 2.5 cm in diameter. Microscopically, chorangioma is identified by a well circumscribed collection of fetal blood vessels supported by scant connective tissue.

## 3. Results

**Figure 1:**
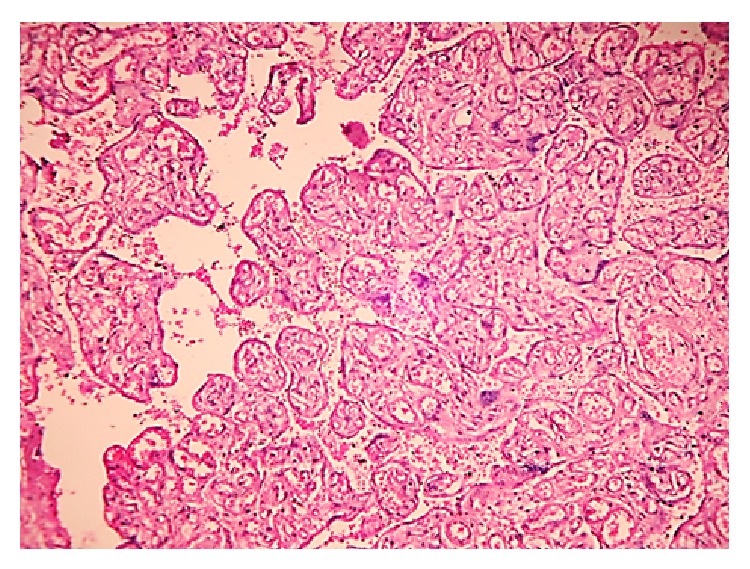
Placental tissue from an anemic mother displaying chorangiosis: H&E; 100x.

**Figure 2:**
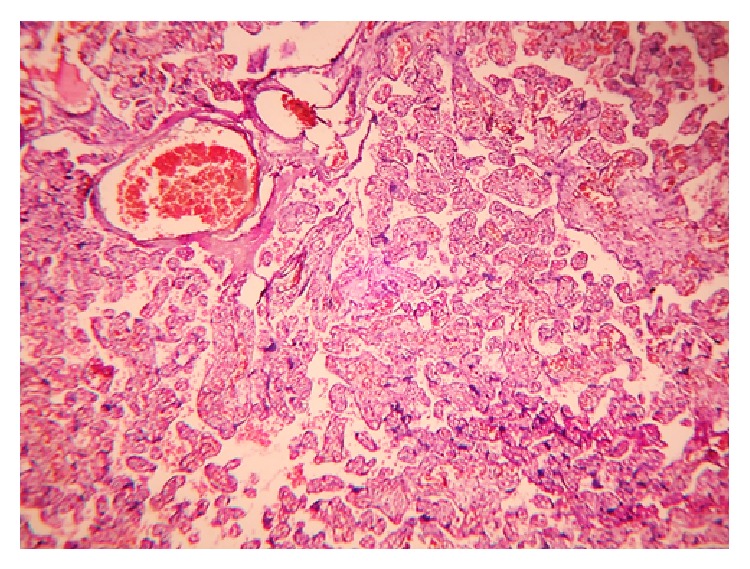
Terminal villi with focal chorangiosis in a placenta from a diabetic woman: H&E; 40x.

**Figure 3:**
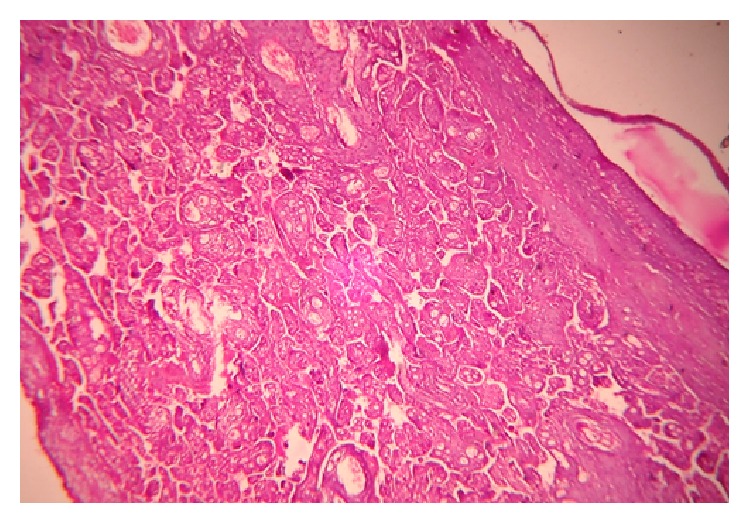
Chorangioma with dilated thin-walled capillaries in the subchorionic tissue: H&E; 100x.

Villous capillary lesions such as chorangiosis (Figures 1 and 2) and chorangioma (Figure 3) were found with increased frequency in the study group compared to the control group placentae (Table 3). In placentae from anemic mothers, the predominant change was increased villous vascularity as seen in 7 (70%) cases. The placentae from mothers with preeclampsia showed decreased vascularity as expected in 6 (60%) cases. The placentae from diabetic and hypothyroid mothers once again showed a dominant increase in villous vascularity in 5 (83.33%) and 3 (60%) cases, respectively. In the control group, 4 (19.35%) cases showed increased villous vascularity, 25 (67.74%) cases had normal vascularity, and 2 (12.9%) cases showed decreased vascularity. Amongst the villous vascular changes encountered in the subset of cases with increased vascularity, chorangiosis was the most common lesion in both groups followed by chorangioma (Table 4) with no evidence of chorangiomatosis in either group (Table 4).

## 4. Discussion

At the beginning of the third trimester mesenchymal villi switch from transforming into immature intermediate villi and start transforming into mature intermediate villi which serve as a framework for the terminal villi which begin to appear shortly afterwards and predominate at term [2]. The terminal villi do not result from trophoblastic proliferation but develop when longitudinal capillary growth outstrips longitudinal villous growth thus causing bulging and protrusions into the intervillous space. The fetal vessels of the terminal villi are therefore represented by capillary loops, parts of which are sinusoidally dilated, arranged such that three to five terminal villi are supplied by an individual coiled capillary loop. This anatomical arrangement significantly reduces blood flow resistance and is completed by the 28th week of gestation, with further increases in nutrient transfer capacity enhanced by increased numbers of villi rather than individual maturation.

The terminal villi of the mature placenta usually contain between two and six capillary vessels, which are sinusoidally dilated so as to occupy the majority of the cross-sectional area of the villus [2]. Three possible deviations from this normal vasculature are encountered, namely, villous avascularity, hypovascularity, and hypervascularity.

Villous capillarization is difficult to interpret as capillaries tend to collapse after delivery. The degree of collapse depends on the mode of delivery, the mode of cord clamping, the time elapsed between cessation of umbilical circulation and fixation, and the composition of fixative. Conclusions concerning villous capillarization must always take potential errors into consideration.

Reduced capillarization of the terminal villous tree is found in slightly immature placentae while complete atrophy of capillaries is seen mostly in placentae associated with IUGR or intrauterine fetal death.

Villous hypervascularity, in which individual terminal villi contain an excessive number of vessels, has been classed as “chorangiosis” by Altshuler who considered that this abnormality could be diagnosed when microscopy with ×10 objective showed 10 villi, each with 10 or more fetal vessels, in 10 or more noninfarcted areas of the placenta [1] and this hypercapillarization was the main feature noted in our study too.

Cases of hypoxic capillarization are most often encountered in maternal anaemia and few cases of IUGR with preserved end-diastolic umbilical flow with or without preeclampsia [3]. It is characterized by numerous but small capillary cross-sections in clusters of aggregated terminal villi. Chorangiosis is a numerical increase of capillaries within the peripheral placental villi and may be an indicator of chronic prenatal hypoxia. Chorangiosis has been reported in pregnancies at high altitude [4] and in severely anemic mothers [5].

Numerous other conditions have been suggested as having an etiologic role in the development of chorangiosis, including maternal, fetal, and placental conditions [6]. The associated maternal conditions include preeclampsia/eclampsia, as already mentioned, diabetes mellitus, drug ingestion, and urinary tract infection. Placental conditions that have been associated with chorangiosis include umbilical cord anomalies, single umbilical artery, abruptio placentae, placenta previa, chorangioma, amnion nodosum, and villitis (rubella virus, cytomegalovirus, syphilis, and* Bartonella *spp. are known to infect and induce proliferation of the endothelial cells) [7]. The fetal factors most commonly associated with chorangiosis are the presence of major congenital anomalies and Apgar scores of less than 5.

Ogino and Redline examined more than 600 placentas and found the villous changes of chorangiosis in about 5% of pregnancies, most frequently in term placentas; they noted that chorangiosis may be associated with maternal diabetes mellitus, placentomegaly, delayed villous maturation, and chronic villitis [8].

This feature needs to be differentiated from excessive nonbranching angiogenesis as seen in severe IUGR with absent umbilical end-diastolic blood flow and abnormally high intervillous oxygen partial pressures as in postplacental hypoxia [7].

Finally, all these need to be differentiated from villous congestion resulting from cord complications and premature rupture of membranes. The presence of overdistended veins in the stem villi, normalcy of villous caliber distribution, loss of plasma between the erythrocytes, extravasations, and signs of hemolysis are helpful in diagnosis [7].

The importance of identifying chorangiosis in placental sections has been emphasized by various authors [9, 10].

In our study, the placentae with chorangiosis were associated with maternal anemia, preeclampsia, gestational diabetes mellitus, and hypothyroidism. Interestingly, few cases of the control group with normal pregnancies also exhibited these changes. This shows that chorangiosis is a normal phenomenon at term and is exaggerated in pregnancies with associated maternal complications.

Chorangioma or placental hemangioma has an incidence of 0.5% to 1% [2] and may vary widely in its macroscopic presentation. Microscopically, it is characterized by numerous blood vessels set in a loose, inconspicuous moderately abundant perivascular stroma containing fibroblasts, macrophages, and scanty fibrous tissue [2]. Angiomatous, cellular, degenerate, and atypical variants may occur. Several placental abnormalities may coexist with chorangiomas. In our study, one of the cases had a placenta with velamentous insertion of cord and another case exhibited placentomegaly with a bulky placenta. The mother in the latter instance was on treatment for hypothyroidism and gestational diabetes mellitus. Chorangiomas, when large, result in complications such as polyhydramnios and premature onset of labour [2].

Chorangiomatosis was first described in 1922 by von Meyenburg [2] and later defined by Ogino and Redline [8] as an excess of vessels within otherwise normal stem villi which does not form an expansile nodular lesion. They further classify the lesion as focal, involving one to five contiguous villous cross-sections, segmental, involving more than five contiguous villous cross-sections, and diffuse or multifocal, involving multiple independent areas of the placenta. Several authors have reported cases of chorangiomatosis and have detailed the clinic-pathologic profile of such cases [11]. In our study, no cases of chorangiomatosis were encountered.

## 5. Conclusion

Based on several observations and studies it is now evident that the development of terminal villi is influenced by the balance of longitudinal growth of mature intermediate villi with that of their capillary loops. Hence any factor that influences angiogenesis, especially hypoxia, whether preplacental or postplacental, can directly influence the number of capillaries occupying a single terminal villus. This reinforces our observation of more instances of chorangiosis in placentae that have suffered significant hypoxic insults due to maternal diseases complicating pregnancy. Our study is unique for the reason that villous vascular change in the form of chorangiosis was unusually found in cases of hypothyroid mothers and even in normal term gestations. Literature based evidence was available for all the subgroups of the study group placentae except hypothyroid placentae. Further studies in this regard are required to establish the importance of such an occurrence.

## Figures and Tables

**Table 1:** Gestational age and birth weight in control group.

Control group (case)	Mother's gestational age (weeks)	Birth weight of infant (kilogram)
1	37	3.4
2	36	3.2
3	38	3.5
4	36	3.5
5	37	3.4
6	38.2	3.7
7	38	3.5
8	36.5	3.3
9	37.1	3.6
10	38	4.0
11	39.2	4.1
12	36.4	3.6
13	39	3.8
14	39	3.5
15	38.2	3.4
16	37	3.4
17	38	3.9
18	39	4.1
19	37.5	3.4
20	36.3	3.4
21	37	3.7
22	38	3.5
23	38	3.6
24	39.1	3.4
25	36.5	3.4
26	37	3.7
27	37	3.5
28	36.3	3.2
29	39	3.9
30	37	3.4
31	38	3.4

**(a):** 

Group I (maternal anaemia)	Mother's gestational age (weeks)	Birth weight of infant (kilogram)
1	36.1	3.0
2	37.4	3.3
3	37	3.0
4	36.5	2.9
5	38	3.2
6	37.2	3.4
7	38	3.3
8	39.1	3.5
9	36.4	3.2
10	37	3.2

**(b):** 

Group II (preeclampsia)	Mother's gestational age (weeks)	Birth weight of infant (kilogram)
1	37	3.1
2	36.1	3.0
3	36.4	3.0
4	38	3.2
5	37	3.1
6	37.1	2.9
7	36	3.1
8	36.2	3.2
9	37.1	3.2
10	38	3.4

**(c):** 

Group III (gestational diabetes mellitus)	Mother's gestational age (weeks)	Birth weight of infant (kilogram)
1	37.5	3.4
2	36.2	3.2
3	38	3.5
4	38.1	3.4
5	37.5	3.4
6	37	3.1

**(d):** 

Group IV (hypothyroidism)	Mother's gestational age (weeks)	Birth weight of infant (kilogram)
1	36.4	3.3
2	37	3.4
3	38.2	3.4
4	37	3.1
5	37.3	3.1

**Table 3:** Villous vascular changes in placentae of control and study groups.

Villous vascularity	Control group	Study group			
	Normal pregnancies number of cases (%)	Group I (maternal anaemia) number of cases (%)	Group II (preeclampsia) number of cases (%)	Group III (gestational diabetes mellitus) number of cases (%)	Group IV (hypothyroidism) number of cases (%)
Decreased	2 (12.9)	1 (10)	6 (60)	—	1 (20)
Normal	25 (67.74)	2 (20)	2 (20)	1 (16.66)	1 (20)
Increased	4 (19.35)	7 (70)	2 (20)	5 (83.33)	3 (60)

Total	31	10	10	6	5

**Table 4:** Spectrum of villous vascular lesions identified in the study and control groups.

Villous vascular lesions	Normal pregnancies	Group I (maternal anaemia)	Group II (preeclampsia)	Group III (gestational diabetes mellitus)	Group IV (hypothyroidism)
Chorangiosis	2	3	1	2	2
Chorangioma	—	1	1	—	—
Chorangiomatosis	—	—	—	—	—
